# Formicidae of the Andaman and Nicobar Islands (Indian Ocean: Bay of Bengal)

**DOI:** 10.1673/031.010.14132

**Published:** 2010-10-06

**Authors:** Prashanth Mohanraj, Musthak Ali, K. Veenakumari

**Affiliations:** ^1^ Central Agricultural Research Institute, P.B. No. 181. Port Blair 744 101, Andamans, India; ^2^ Department of Entomology, University of Agricultural Sciences, GKVK, Bangalore-560065, India; ^3^ Present address: Project Directorate of Biological Control, P.B. No.2491, Hebbal, Bangalore-560024, India

**Keywords:** Ants, Andamans, Nicobars

## Abstract

Ants on the Andaman and Nicobar Islands, India were surveyed. These collections doubled the number of ant species recorded from these islands (from 59 to 125). Records include five endemic species, but no endemic genera. The surveys were fairly superficial, and it is likely many species remain to be discovered on these islands.

## Introduction

It has been 3 decades since McVean (1976) evaluated the status of zoological studies on the Andaman and Nicobar islands (India) and observed that many insect species needed to be studied as they have been “hitherto neglected [in studies on] the natural history of these islands”. Along with a number of other insect groups, the Formicidae have continued to remain largely neglected, even after this observation was made. The only papers on the Formicidae of these islands during this period were by Chhotani and Maiti (1977) and Tiwari and Jonathan (1986a,b). Together these reports added a mere 11 species to the ant fauna already known from these islands, of which nine were new records and two were new species.

Mayr (1865) was perhaps the first to describe Formicidae from these islands, mainly from the Nicobars, based on collections made by the Austrian frigate *Novara.* Years later Forel (1903) listed a total of 39 species of ants, which included species from Mayr (1865), as well as the descriptions of three new species. The new descriptions included two species from the Andamans and one from the Nicobars. In the same year, Bingham (1903), while dealing with the ants of the Indian subcontinent in the Fauna of British India series, mentioned a mere two species from the Nicobars and none from the Andamans. Emery (1911, 1912, 1921, 1922) made some references to ants from these islands. The collection of Dr. N. Annandale, made during November and December 1923 from Mount Harriet (S. Andaman), was described by Mukherji and Ribeiro (1925). This included 15 species, of which two were recorded at the generic level only. Chhotani and Maiti (1977), working on another collection, the Zoological Survey of India that was collected between February and April 1964, reported 15 species of ants, of which 10 were new records. Tiwari and Jonathan (1986a, 1986b) described two new species, one each from South Andaman and Great Nicobar. These species belong to genera that were not known previously from these islands. In his revisionary studies, Bolton (1987) added one more species to the ant fauna of these islands. In short, a total of 59 Formicidae species (of which, four were identified to the generic level only) were recorded from these islands between 1865 and 1987. No papers on the ants of these islands have appeared since then.

## The Island Setting

### Area

**Figure 1.**
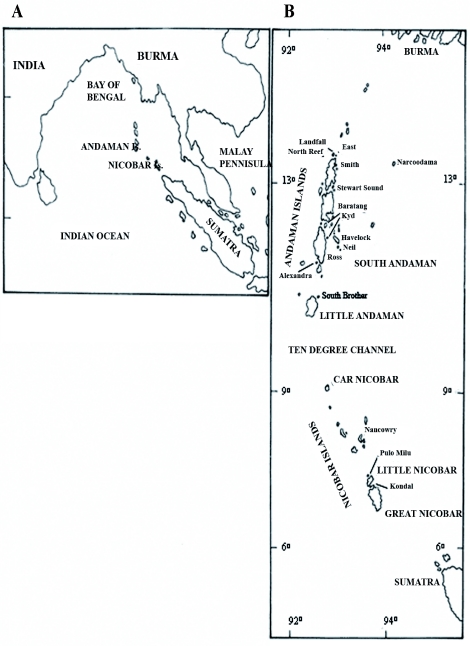
(A) Location of the Andaman and Nicobar Islands in the Bay of Bengal, Indian Ocean. (B). Andaman and Nicobar Islands showing the islands from where ants were collected (Maps not drawn to scale). High quality figures are available online.

The Andaman - Nicobar chain of islands situated between 6° 45′ - 13° 30′ N and 92° 94° E in the Bay of Bengal, Indian Ocean (Figure 1A, B) consists of 572 islands, islets and rocks (Anonymous 1986). The Andaman islands (6408 km^2^), which are more than three times larger than the Nicobars (1841 km^2^), are separated from the latter by the 150-km-wide Ten Degree Channel. In turn, each island group is fragmented by straits and channels of varying widths and depths.

### Tectonics and sea level changes

These are true oceanic islands lying along the 6000 km long Sunda Arc, which extends west from the island of Sumatra to Burma in the north and marks the zone where the Indian-Australian plate is being subducted beneath the Eurasian plate (Moore et al. 1980; Curray 1989). Subduction, which is presumed to have commenced about 130 MYBP following the breakup of Gondwanaland in the early Cretaceous, resulting in the formation of oceanic ridges that were uplifted to their current elevation as two arcs in the late Eocene or early Oligocene times (Curray et al. 1979). The outer arc ridge emerges intermittently above sea level as the Andaman-Nicobar islands, while the Barren and Narcondam islands are the emergent peaks of the submarine ridge that forms the inner volcanic arc (Hamilton 1979).

It is thought that in the Pleistocene, when sea levels were lower, there was no dry land connecting the islands to any of the adjacent land masses (Ripley and Beehler 1989). There is also no evidence for the existence of an earlier land bridge, even during Tertiary times (Nassig et al. 1996). During times of sea level lowering, however, the sea separating the Andaman islands from Burma was much narrower and shallower than that separating the southern Nicobars from Sumatra or the Malay Peninsula (Ripley and Beehler 1989).

### Topography

The surface of the majority of the islands is irregular and hilly, with many narrow valleys. The hills, which follow the general direction of the islands, are oriented in the north-south direction, and from which arise numerous spurs and ridges that branch out in all directions. The hills on the east are higher than those on the west, with Saddle Peak (732 m) in North Andaman being the highest point in the Andamans and Mount Thuillier (642 m) in Great Nicobar is the highest in the Nicobars (Anonymous 1994).

### Climate

The islands experience a tropical maritime climate that is strongly influenced by the Indian Ocean. They receive over 3000 mm of rainfall between May and December during both the South-West and the North-East monsoons. The mean minimum and mean maximum temperatures vary between 23° C and 30° C with maximum temperatures of about 34° C attained in April. High humidity prevails throughout the year, ranging from more than 60 to about 90 percent.

### Vegetation

The islands are densely wooded from the water's edge to the tops of the hills, except in those areas initially cleared by the European colonial powers and later by settlers from the Indian mainland. Native vegetation has been replaced to varying degrees by settlements and introduced plants (intentionally for cultivation and accidentally as weeds) on all of the 38 inhabited islands. Over the years, this has led to near extinction of the Andaman Giant Evergreen forests, one of the seven major forest types characterized by Champion and Seth (1968) as occurring on these islands.

## Materials and Methods

Ants were collected from cultivated and uncultivated sites, both from the ground and the vegetation. No special methods were used. Ants were located visually while walking randomly, and they were collected in alcohol tubes using a camel hair brush/forceps. T wigs were broken to collect nesting arboreal ants.

### Collection localities

Ants were collected from 14 islands in the Andamans and three in the Nicobars (Table 1) at various times between 1989 and 1998. It was, however, on the island of South Andaman that over 80% of the time was spent collecting ants. Only 13% of the time was spent in the Nicobars, with most of this time (over 98%) spent on the island of Great Nicobar. The islands from where ants had been collected earlier but which the authors were unable to visit were South Brother and North Reef in the Andamans and Pulo Milu in the Nicobars (Mayr 1865; Forel 1903; Mukherji and Ribeiro 1925; Chhotani and Maiti 1977).

The ants collected by the first and third authors were identified by Musthak Ali, the second author.

## Results and Discussion

This study raises the total species of ants known from these islands to 125, from a previous total of 59. Only 37 of the 59 previously reported species of ants were collected (Table 2). The 125 species of ants listed here belong to 41 genera. While 10 genera (*Anochetus, Platythyrea, Cerapachys, Bothriomymex, Dolichoderus, Gnamptogenys, Hyoponera, Podomyrma, Pristomyrmex*, and *Technomyrmex*) were recorded for the first time from these islands, seven genera (*Myopopone, Paratopula, Vollenhovia*, *Liomyrmex, Metapone, Acropyga*, and *Echinopla*) found in the earlier literature were not encountered. Of these seven genera, all but *Paratopula* and *Liomyrmex* were collected from the Nicobar islands, where this survey was limited.

**Table 1:**
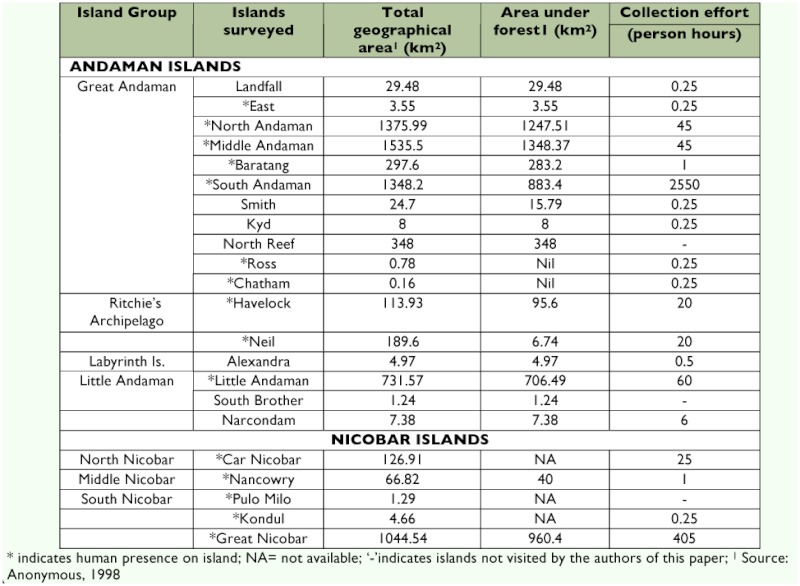
.Islands of the Andamans and Nicobars from where ants were collected.

The fact that some Formicidae previously recorded from these islands were not collected does not imply that they have gone extinct. It only denotes that more intensive collections have to be made when most, if not all, of these species are likely to be found.

*Echinopla, Myopopone, Odontoponera*, and *Philidris* are genera with Malesian (or Indoaustralian) + Australasian distribution that find their western limit on these islands. Not only was *Echinopla* not found on these islands, but there is also some doubt about the occurrence of the genus on these islands. The lone species of the genus was reported from the southern Nicobar islands as *Echinopla senilis* by Mayr in 1862. This species was later considered a variety of *E. lineata* Mayr by Emery (1896), who, however, did not furnish any reasons justifying the change. In all probability, no one has ever seen this species after 1862 (Baroni Urbani 1997 *In litt.*).

**Table 2.**
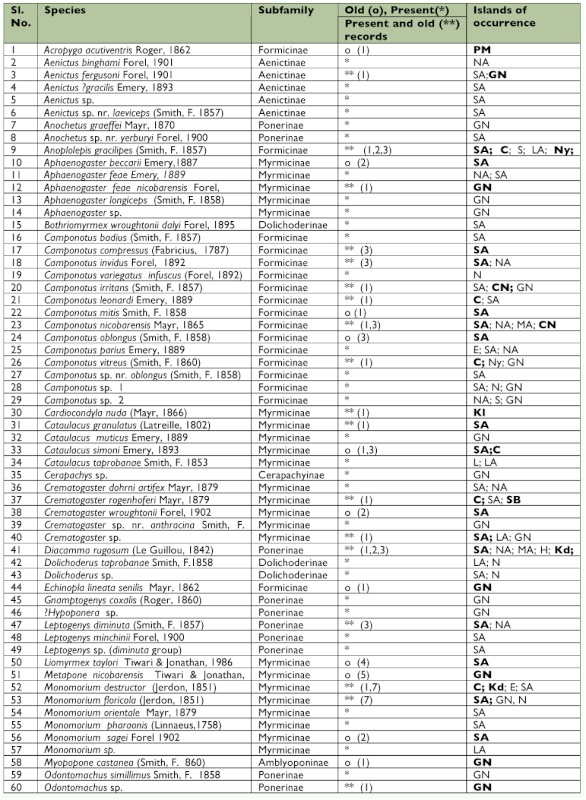
Preliminary list of ants of the Andaman and Nicobar islands

**Continued.**
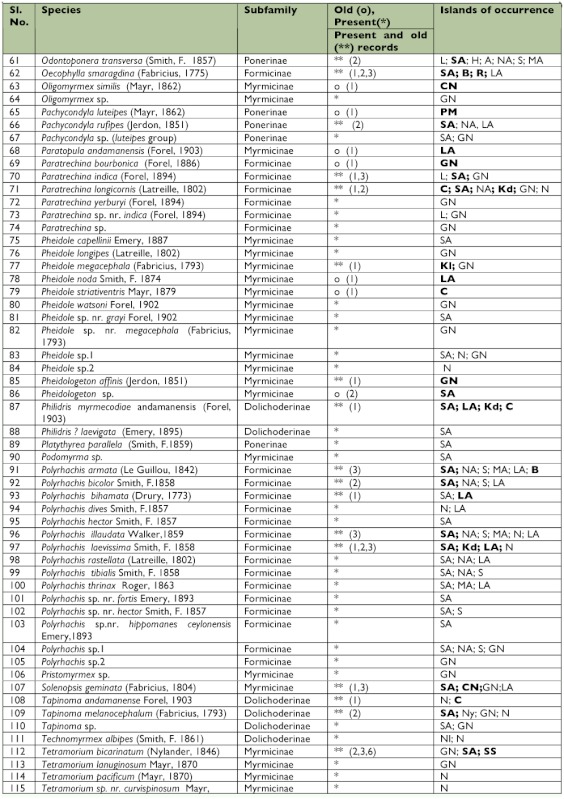

**Continued.**
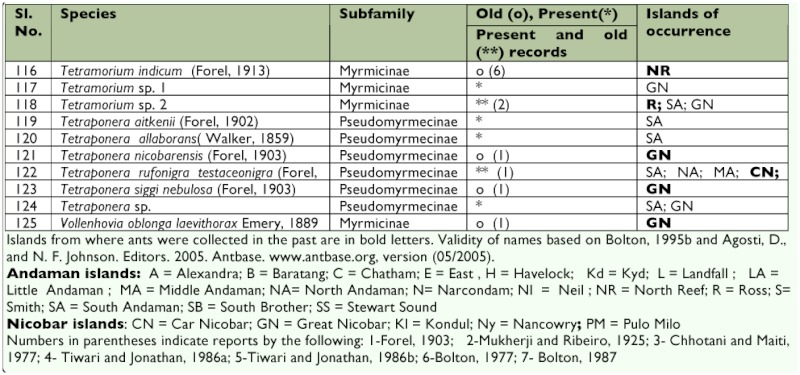

*Cerapachys*, Tapinoma, Acropyga, *Camponotus, Paratrechina, Crematogster, Monomorium, Pheidole, Solenopsis*, and *Tetramorium* are 10 of the 15 most widely distributed genera (i.e., those that are found in all the 8 Zoogeographic regions of the world) (Bolton 1995) that are found on these islands.

*Hydnophytum formicarum* Jack (Rubiaceae) and *Dischidia major* (Vahl.) Merr. (Asclepiadaceae) are the two myrmecophytes that are found associated with species of *Philidris* on these islands. Also, *Cataulacus* sp. was found nesting in *Dishcidia* on the Andaman Islands.

*Anoplolepis gracilipes* and *Pheidole megacephala* are among the world's worst invasive ant species (Global Invasive Species Database, http://www.issg.org/database). Both were found on these islands in the early twentieth century (Forel 1903). It is important to note that Forel's study appeared about fifty years after the British had occupied these islands. *A. gracilipes* is widely distributed in the Afro-Tropical region, and it is a wellknown tramp species. It is known to have caused extensive environmental damage in island ecosystems especially in Hawaii, Seychelles, Zanzibar, and Christmas Island. Along with *P. megacephala*, these species pose a serious threat to the native invertebrate fauna of these islands.

It is interesting to note in this context that 10 species of ants were intercepted in 18 shiploads of timber transported between 2006 and 2009 from Malaysia and Myanmar to ports along the west coast of India. These were *Anoplolepis gracilipes, Pheidole sp., Diacamma rugosum, Camponotus* *compressus, Polyrhachis rastellata*, *Rhoptomyrmex wroughtoni, Aphaenogaster sp., Paratrechina longicornis*, and *Pheidologiton diversus* (Manickam, personal collection).

Considering that the Andaman islands have three times more area than the Nicobars, they should also be expected to harbour a richer biota than the latter island group. In fact, the data from this survey seem to reflect this trend. However, since the collection efforts were grossly uneven between the two island groups, with greater effort expended on the Andamans, this cannot be taken as a true reflection of greater species richness in the Andamans than in the Nicobars. To arrive at a truly representative picture of the relative diversities of Formicidae between the two island groups, more intensive collections will have to be made.

With transoceanic dispersal capabilities only marginally poorer than bats and better than many insect orders (like Trichoptera, Isoptera, etc.), ants are among the most successful early colonists of islands (Zimmerman 1948). This, and the fact that no studies have so far been focused exclusively on the ants of these islands, makes it almost certain that many more genera/species await discovery, particularly in the leaf litter and arboreal habitats. It is, therefore, imperative that studies are initiated on the ants of these islands before habitat destruction leads to the elimination of many species even before they are discovered.

Voucher specimens have been deposited at the University of Agricultural Sciences, GKVK, Bangalore and the Department of Entomology, Indian Agricultural Research Institute, New Delhi, India.
